# Lost in explanation: internal conflicts in the discourse of ADHD psychoeducation

**DOI:** 10.1186/s12888-022-04327-x

**Published:** 2022-11-08

**Authors:** Myrte J. M. van Langen, Rebeka Szőke, Dominique N. J. Rijkelijkhuizen, Sarah Durston, Branko M. van Hulst

**Affiliations:** 1 University Medical Center Utrecht, Utrecht, The Netherlands; 2 Doctoral School of Biology, Institute of Biology, ELTE Eötvös Loránd University, Budapest, Hungary; 3 Department of Psychology, Utrecht University, Utrecht, The Netherlands; 4 LUMC Curium - Child and Adolescent Psychiatry, Leiden University Medical Center, Leiden, The Netherlands

**Keywords:** Attention Deficit Hyperactivity Disorder, ADHD, Psychoeducation, Discourse Analysis

## Abstract

**Background:**

Psychiatric classifications are understood in many different ways. For children with ADHD and their parents, psychoeducation is an important source of information for shaping their understanding. Moreover, psychoeducation is often taken by children and parents to represent how their story is understood by the therapist. As a result, the way psychoeducation is formulated may affect the therapeutic alliance, one of the most robust mediators of treatment outcome. In addition, psychoeducation may indirectly influence the way we understand psychological differences as a society.

**Methods:**

To better understand how the classification ADHD is given meaning through psychoeducation, we analyzed 41 written psychoeducational materials from four different countries; the USA, UK, Netherlands and Hungary.

**Results:**

We identified five patterns of how the materials construct the discourse on ADHD. Notably, tension between biomedical and psychosocial perspectives resulted in conflict *within* a single thematic stance on ADHD as opposed to a conflict *between* parties with a different vision on ADHD. There were only few differences between countries in the way they constructed the discourse in the materials.

**Conclusions:**

These conflicts cause confusion, misrepresentation and decontextualization of ADHD. Ultimately, for those diagnosed with ADHD and their parents, conflicting information in psychoeducation materials may hamper their ability to understand themselves in the context of their difficulties.

**Supplementary Information:**

The online version contains supplementary material available at 10.1186/s12888-022-04327-x.

## Background

Psychiatric classifications are terms that refer to clusters of symptoms [1, 2]. Such classifications have extensive impact, as they are often taken by a person to represent how their story is understood by the therapist [3]. As a consequence, the way a classification is given meaning, including through psychoeducation, may affect the therapeutic alliance. This therapeutic alliance is well known to be the most robust mediator of treatment outcome [4–8]. Moreover, the impact of psychiatric classifications stretches well beyond a strict healthcare perspective [9–11]. Psychiatric classification and concurrent psychoeducation may indirectly influence the way we understand psychological differences in society.

Psychoeducation on ADHD is a special case, as the ongoing debate on ADHD exemplifies tensions between biomedical and psychosocial perspectives on mental health [12–14]. Notably, these two perspectives together form the basis of one of the most widely accepted approaches to mental health: the biopsychosocial model [15]. The biomedical perspective considers ADHD to have a biological cause, and understands it as a heritable, persistent neurodevelopmental disorder [16–19]. The psychosocial perspective understands ADHD as a dynamic outcome of how an individual interacts with his or her individual circumstances, including at home and school or work on a day-to-day basis [20, 21]. These perspectives are not mutually exclusive. However, the way they are integrated and represented in psychoeducation is likely to impact both the therapeutic alliance and societal ideas on inattention and hyperactivity. Yet very little is known about how the classification ADHD is given meaning through psychoeducation.

Studies assessing biomedical and psychosocial perspectives in various texts on ADHD have mostly applied discursive analytic techniques. They start from the crucial assumption that information provided in psychoeducational materials mirrors the discourse around ADHD, while simultaneously constructing that same discourse [22–24]. Previous studies have often found the biomedical perspective to be overrepresented. In Dutch youth information books on ADHD, text on both psychosocial and biomedical perspectives was included [20]. However, biomedical text elements were overrepresented in two ways, with greater variety in the information offered (eight biomedical versus five psychosocial categories) and more instances of it being offered (207 biomedical versus 91 psychosocial text elements) [20]. A similar discrepancy was reported for English language websites on ADHD: 96,5% of websites were found to emphasize bio-genetic over psychosocial causes [14]. For French media and tv programs [25, 26], findings were more nuanced: although French tv-programs did over-represent biomedical perspectives, popular and professional literature were found to often combine both perspectives. Furthermore, one study found psychosocial repertoires to be overrepresented in UK newspaper articles (72 psychosocial versus 16 biomedical repertoires) [27]. Whereas, these studies have investigated the extent to which biomedical and psychosocial perspectives were represented, they did not analyze the integration of these perspectives. A study by Erlandsson et al., [28] did address this complex integration of information: the authors analyzed a single document on ADHD [29] and found a clear bias towards biomedical explanations, as well as a focus on expert knowledge and circular reasoning.

To better understand how the classification ADHD is given meaning through psychoeducation, we analyzed 41 written psychoeducational materials from four different countries; the USA, the UK, the Netherlands and Hungary. Our primary aim was to analyze how the *explanatory framework* of ADHD is constructed through language use in these psychoeducational materials. We therefore carried out an exploratory study of patterns in how ADHD is framed and contextualized. In addition, we performed a cross-national analysis. Based on literature showing cross-cultural variation in the understanding and interpretation of ADHD [30, 31], we hypothesized that we would find differences between countries in how psychoeducational materials constructed the discourse on ADHD, even though they operate from the same diagnostic handbook (the DSM-5). Overall, this paper aims to describe prominent discursive patterns in psychoeducational materials on ADHD, differences between countries in this discourse and to discuss the impact of the discourse on how stakeholders may understand and interpret information on ADHD.

## Methods

### Procedure

In this discourse analysis, we assessed American English, British English, Dutch and Hungarian psychoeducational materials on ADHD. We selected 8–12 materials in each language and assessed the commonalities and variation in how these materials constructed the discourse on ADHD.

### Selection Psychoeducational materials

First, we performed an online scoping search to determine appropriate inclusion and exclusion criteria. Our psychoeducational materials reflect a broadly selected set of psychoeducational texts, including webpages, downloadable PDF-files with psychoeducational text and downloadable flyers. We performed a broad internet search using the following search terms or combinations thereof: ADHD, Attention Deficit Hyperactivity Disorder, psychoeducation, material, flyer, and diagnosis. Supplemented with a search term matching the appropriate language area: Great Britain, UK, United States, US, Netherlands, Dutch, Hungary, Hungarian. Search-terms were translated to search for Dutch and Hungarian materials.

We used the following inclusion criteria:

- Standalone materials (materials that were not solely part of a broader psychoeducational program or therapeutic manual).
- Materials that were freely accessible online
- Materials written in American English, British English, Dutch and Hungarian or translated into these languages
- Materials written for parents of children with an ADHD classification

We used the following exclusion criteria:

- Materials written by the same author and/or published by the same organization
- Materials over 5000 words (approximately 10 pages - to keep data analysis manageable)

We subsequently selected the first 8–12 materials in each language. This selection resulted in a convenience sample adapted to online-searchability. This sample is not representative of all psychoeducational material on ADHD but rather roughly mirrors the likelihood that materials will be found online by parents.

### Data processing

We collected and stored all psychoeducational materials in the original lay-out. We then transcribed materials into plain text files and entered them into a software package for qualitative data analysis (Nvivo 12). Descriptive data was summarized for each of the selected materials (see Additional File 1), including: type of document, word count, author and/or publishing organization, year of publication and intended audience.

### Discourse analysis

Discourse analysis was performed by three independent raters (RS, DR & MvL). A detailed overview of our analysis plan is provided in Additional File 2. First, we conducted a practice analysis on two independent Canadian materials. Second, all researchers rated the UK materials simultaneously in order to further standardize our working procedure. Finally, we rated the US (DR), Hungarian (RS) and Dutch (MvL) materials independently. All analytical steps were completed together for the British English materials and independently for the other materials.

To familiarize ourselves with the data, we read each of the materials four times. We kept notes and held discussion meetings about our findings. We then coded the data in three steps. First, we strictly coded the content of the material to gain insight into the information provided. Second, we carried out a critical interpretation of the data: we coded how the discourse on ADHD was constructed and how implicit ideas and understanding of ADHD were manifest in the text. Third, we coded for language use and phrasing in the text and selected relevant excerpts. After coding every two materials we discussed our findings. When coding was complete, we held a number of conclusive discussion sessions. We discussed our findings and settled on the interpretation of major themes, discursive patterns and associated text excerpts. We then analyzed the data again, coding for these predefined discursive patterns, to reassess our results and generate a comprehensive list of relevant text excerpts.

## Results

In the sections below, we describe the results of our analysis of 41 psychoeducational materials from the United Kingdom (10), the Netherlands (10), Hungary (10) and the United States (11). Our results are presented in three sections: first, we discuss the content and major themes in the materials. Second, we discuss the five discursive patterns we found in the materials. Third, we present a cross-cultural comparison of the discursive patterns in the materials.

### Thematic content

In the first step of the analysis, we examined the content of the materials. We identified three themes: (1) Definition and Diagnosis, (2) Causes and Risk Factors, and (3) Treatment and Prognosis. Below we describe these themes to introduce the information provided in the materials.

The first theme was *Definition and Diagnosis*. Most materials started with a description and introduction of ADHD. ADHD was described as a chronic, neurobiological, neurodevelopmental, psychiatric, behavioral disorder or condition. It was described as a real disorder recognized internationally by professionals. Children were said to receive a diagnosis if they exhibited symptoms of ADHD. The materials discussed a wide variety of symptoms, many of which could be categorized as inattention, hyperactivity or impulsivity. Materials often mentioned that symptoms should occur in multiple environments for more than six months, should start at an early age and should cause impairment in everyday life. They also noted that a diagnosis is helpful and necessary for adequate and effective treatment. They stated that ADHD is very common among children and adolescents and it is diagnosed more often in boys than in girls. They also described that ADHD often co-occurs with comorbid disorders and additional problems.

The second theme was *Causes and Risk Factors*. Most materials provided a thorough description of the etiology of ADHD. This etiological description usually discussed both neurobiological and environmental factors. Neurobiological factors mentioned included genetics and differences in brain regions, brain functioning, or neurotransmitter systems. Environmental factors mentioned included premature birth, pregnancy complications and substance abuse during pregnancy, as well as parenting styles and family stress. Neurobiological factors usually took precedence over environmental factors in the way they were described. Many materials explicitly mentioned that environmental factors may contribute to, but do not cause, ADHD. Materials often referred to neurobiological factors as causes and environmental factors as risk factors.

The third theme was *Treatment and Prognosis*. Most materials mentioned that there is no cure for ADHD. However, most indicated that prognosis improves greatly with adequate treatment. Some materials mentioned that children can outgrow ADHD while other materials stated that affected children will have ADHD for life. Materials discussed a large variety of treatment options. These included behavioral treatments, parent training, environmental adaptations as well as a number of pharmacological options. Materials varied in how important they deemed behavioral treatment, environmental adaptations and pharmacological treatments to be. Most materials agreed that treatment prevents children from derailing and helps them to reach their full potential. Most argued that without adequate guidance, children would experience serious negative consequences of their ADHD.

### Discursive results

In the second step of the analysis, we examined how the discourse on ADHD was constructed within these three themes. We identified five discursive patterns that are described below. In addition, we found that four out of five of these patterns contained an element of internal conflict. We defined internal conflict as a situation where different elements of the same explanatory framework are in (apparent) disagreement with each other. The term ‘internal’ therefore refers to a conflict that is present within an overarching explanatory framework. Sometimes this was even present *within* a single psychoeducational document, but this conflict was certainly present across materials with similar thematic stances on ADHD. These conflicts were usually not explicit in the materials, but rather implicit in the information provided. To illustrate the discursive patterns, we have added exemplary quotes for each of the discursive patterns. An extensive list of exemplary quotes is provided in Additional File 3.

#### Pattern 1: cause versus consequence

We found that ADHD was presented as both a cause and a consequence of the same phenomenon, sometimes even in the same material. This was particularly noticeable for the themes *Definition and Diagnosis*, and *Causes and Risk Factors*. ADHD was described as a name given to a cluster of symptoms and simultaneously as the cause of those same symptoms. An example is given below, where ADHD is said to cause neurobiological differences, and to be caused by those same neurobiological differences.

*“Attention deficit hyperactivity disorder is a condition, which affects those parts of the brain which control attention, impulses and concentration (a neurobiological condition).” -* UK Material 11

*“ADHD is thought to be caused by an imbalance of two neurotransmitters, dopamine and noradrenaline, which are believed to play an important role in the ability to focus and pay attention to tasks.” -* UK Material 11

#### Pattern 2: uncertain complexity versus certain simplicity

We noted that ADHD was often presented as a complex and multifactorial disorder that is not yet fully understood. Yet the information on causes and risk factors was simple, certain and clear, suggesting that ADHD is in fact well-understood. For example, materials mentioned that ADHD is complex, yet they would often go on to delineate simple categories that cause (neurobiology, genetics) or modulate (environment) ADHD. In a similar vein, materials often mentioned that the causes of ADHD are unknown, but just as often explained the causes of ADHD. The uncertainty and the intricacy of interplay between these factors were left out of the explanations. As such, materials constructed a simplified image of ADHD that contradicted the complexity they acknowledged elsewhere.

*“Scientists have not yet identified the specific causes of ADHD.”* - US material 2

*“ADHD is a disorder in certain areas of the brain and is inherited in the majority of cases. It is not caused by poor parenting or a chaotic home environment”* – US material 7

#### Pattern 3: normality versus abnormality

We noted that materials both normalized and abnormalized ADHD. This pattern was particularly evident in the themes *Definition and Diagnosis* and *Treatment and Prognosis*. In these themes, behavior related to ADHD was referred to as a common variation of normal childhood behavior. Materials noted that every child displays these behaviors to some extent during their development and these behaviors should be considered normal. Yet, at the same time, the psychoeducational materials stressed that ADHD is a real and serious disorder. It was described as a distinct category of behavior that has a major impact on a child’s life. These abnormal behaviors should be dealt with adequately. Both of these realities seem to exist simultaneously across materials, without their inconsistencies being acknowledged.

“*Its core symptoms are hyperactivity, impulsivity and inattention. These common childhood behaviours occur on a continuum from normal to abnormal. It can be very difficult to judge what ‘normal’ behaviour is in children; therefore when evaluating children for ADHD, many doctors try to assess the degree of impairment caused by these behaviors.”* - UK Material 10

*“The recognition of ADHD as a serious medical condition continues to grow by physician groups and government health agencies around the world.”* – UK Material 3

#### Pattern 4: specificity versus generality

We identified a pattern in the theme *Definition and Diagnosis* where materials specifically defined what ADHD entails and simultaneously provided a general and extensive list of (associated) symptoms. The initial definition mostly adhered to DSM-5, mentioning three categories: inattention, hyperactivity and impulsivity. However, in a subsequent description of ADHD, materials included such a wide variety of symptoms, that this description broadened and blurred the definition. We have illustrated this pattern in Table 1 by providing a list of all ADHD-related symptoms mentioned in the materials from the UK. The result is a list of symptoms of which one or more will be experienced by many, if not all, children while growing up.

**Table 1:** *List of all ADHD-related symptoms mentioned in the UK Materials*

Impulsiveness Hyperactivity/Being overactive Inattention/ short attention span Restlessness Fidgety Full of energy Loud and Noisy Continuous chatter/Talking excessively Talks when others are talking Doing things repeatedly without thinking Finding it hard to wait their turn in games or a queue Interrupting others in conversation or in play Hardworking Persevere at tasks Eager to try new things Appear overly forgetful Distracted Disorganized	Academic underachievement Unable to listen or concentrate Slow to start tasks Struggle to finish tasks and often don’t Creative Intelligent Determined Good at problem-solving Lack of coordination Lack of social skills/social clumsiness Learning difficulties/disabilities Autism Conduct disorder/Oppositional defiant disorder Anxiety Depression Dyslexia, Language problems Difficulties with handwriting	Neurological problems (tics or epilepsy) Can’t sit still, walks, runs Can’t do any one thing for very long Climbs around when others are seated Daydreaming /seeming to be in another world Sidetracked by what is going on in surroundings Mood swings Being careless Making too many mistakes at school Making silly or careless mistakes Disruptive in play Always on the go Often lose their belongings Lacking attention to details Being impatient Poor self-esteem/feeling insecure Clumsiness Temper outbursts

*“ADHD is a well-defined clinical condition. All the major medical authorities recognise it, including the World Health Organisation and the American Psychiatric Organisation.”* – UK Material 7

#### Pattern 5: necessity of the expert view

Materials constructed ADHD as a very impactful, serious, negative and dangerous disorder that required proper treatment. It was described as a largely individual and usually biological problem: something in the child’s biology causes problematic behavior. In addition, materials described that the consequences of ADHD stretch well beyond the individual child. These consequences affect not only the child’s development, but also parents, siblings, peers, school and even society at large. In all, ADHD was explained as an individual problem that has far-reaching societal consequences. Due to this extensive impact, materials emphasized the necessity for children to receive proper care and treatment, for which expert knowledge is required in every step of the process. Expert status was assigned to professionals and clinicians. Materials paid limited attention to the experiences and knowledge of children and their parents. Children with ADHD were usually not mentioned as active agents in the process and were not mentioned in the communication about their experiences. Likewise, the child’s positive characteristics received little attention. In all, psychoeducation materials did not attribute a form of expert-status to parents or children (although parent were assigned a more proactive role in American-English psychoeducation, see the section below on *Agency of Parents*).

*“The knock-on effects of poorly managed or even unidentified ADHD, most notably the potential decline into the criminal justice system, highlight that early intervention is essential.”* - UK Material 3

*“Left untreated, ADHD in some children will continue to cause serious, lifelong problems, such as poor grades in school, run-ins with the law, failed relationships, and the inability to keep a job*.” – US Material 4

### Differences between countries

Overall, we found many more similarities than differences between countries. All of the patterns discussed above were present in materials from all countries. In this section we will briefly discuss two prominent differences we did find.

#### Variability in etiological explanations

We identified a difference in the discourse on ADHD etiology across countries. Dutch and Hungarian materials represented different perspectives or different ‘explanatory frameworks’, whereas materials in American and British English started from a more homogeneous, neurobiologically oriented perspective. In Dutch, some materials did not discuss etiology at all; some framed ADHD as entirely biological, while others described many different causal and risk factors. These included a variety of neuropsychological profiles, biological maturation, classroom pressure, and the impact of the direct and indirect environment. Hungarian materials were equally variable and in addition introduced more controversial hypotheses. Some mentioned how our current “hyperactive society” shapes ADHD and how trauma, stress or toxins can contribute to its development. UK materials were relatively consistent. They usually discussed three distinct factors; genetics, neurobiology and environment. Genetic and neurobiological factors were presented as causes of ADHD, whereas environmental factors were presented as risk-factors. Numerous environmental factors were mentioned, including perinatal factors, bad parenting and family stress. In US materials, ADHD was usually explained as a genetic disorder that “runs in families”. ADHD was framed as a genetic disorder that would subsequently impact the neurobiology of the child.

#### Agency of the parents

We identified a discursive pattern on parental agency in US materials, distinct from those in other materials. Specifically, parents were actively addressed as important agents in diagnosis and treatment. Parents were advised to read up on ADHD before going to a clinician to get help. Similarly, they were encouraged to actively manage their child’s care and ensure that different parties communicate properly. To this end, US materials point to a law that gives parents and children the right to treatment and additional support. In the other countries, parents were assigned a much less proactive role, especially in the diagnostic process. Contacting an expert was usually recommended as a first step. Parents were told to rely on the expert throughout. With regard to treatment, a number of materials did emphasize the importance of parents and teachers. After learning more about the disorder, they would become “the key” to success.

## Discussion

We carried out a discourse analysis of 41 psychoeducational materials on ADHD from the US, the UK, the Netherlands and Hungary. We explored how the explanatory framework of ADHD is constructed through the use of language. The materials contained a number of internal conflicts in how ADHD was framed and contextualized. Notably, these conflicts remained unaddressed in the documents. Conflicts arose from tension between 1) *cause* versus *consequence*, 2) *uncertain complexity* versus *certain simplicity, 3) normality* versus *abnormality* and 4) *specificity* versus *generality*. In addition, there was a clear pattern of the materials emphasizing 5) *the necessity of the expert view*.

By and large, we did not confirm our hypothesis of cross-cultural differences in how materials constructed the ADHD discourse. However, we did identify two differences between countries in the discourse on ADHD: we found differing etiological preferences and differing preferences for the agency of parents across countries. Here, American-English and British-English materials favored more straight-forward biomedical etiological explanations, while Dutch and Hungarian materials were more likely to include other, environmental explanations. Furthermore, American materials put greater emphasis on the agency of parents than materials from other countries. These differences are likely to reflect differences in national perspectives on mental health [30–33], such as legal differences in the right to care. Overall, however, we found that the similarities in the discourse on ADHD from different countries were much greater than the differences between them. This may well be the consequence of increased global discourse on classifications in general, and DSM in particular [34–36].

We found internal conflicts in the discourse on ADHD, across psychoeducational materials from four different countries. Such conflicts may have a number of consequences. One possible consequence is that children diagnosed with ADHD and their parents might be confused. One of the main aims of psychoeducation is to help children and parents better understand their problems and subsequently promote better coping [37–39]. Yet, if the information provided is conflicting, children and parents may well be left with incoherent integration of the information provided and feel confused as to how to understand themselves or their children. Subsequently, this could affect expectations of coping, recovery and future development [13, 40–43].

A second possible consequence stems from the conflict between *uncertain* complexity and *certain simplicity* in psychoeducational materials. Materials often stated that the causes of ADHD were complex and unknown. Yet, in the simplified information they subsequently provided on causes and risk factors, they omitted details and nuance, nearly always in favor of biomedical causes. One example of such a misrepresentation is materials stating that neurobiological research has shown indisputable and consistent differences between children with and without a diagnosis [44, 45]. However, these differences referred to are only found at a group level and the effect sizes are very small [46, 47]. As such, it is not an indisputable fact that an individual child with a diagnosis differs neurobiologically from a child without a diagnosis. Statements suggesting otherwise can lead parents to believe (and communicate) that their child’s brain is different from that of their peers. Yet, a more nuanced interpretation of the neurobiological literature would be that the likelihood of an actual difference is small at the individual level. As such, parents may conclude from the educational materials that the causes of ADHD are definite and conclusive, while they in fact are not.

A third possible consequence stems from the conflict between *cause and consequence.* We found a form of circular reasoning where ADHD was described as a term given to a cluster of symptoms and, simultaneously, as the cause of those symptoms. This finding is a replication of earlier studies describing this process [28, 44, 45, 48, 49]. Naming ADHD as a cause of symptoms implies that problems lie with or within the child with ADHD and leaves little space for exploring the context in which problematic behaviors occur [13, 21, 45, 50]. This decontextualization is reflected in the language we use to discuss ADHD. For example, Statements such as ‘*ADHD is part of the child’s make-up and doesn’t suddenly appear out of the blue – UK Material 7*’ underline how we individualize ADHD.

Three of the internal conflicts we have described, relate directly to tensions between the biomedical and psychosocial perspectives. In the biomedical framework, defining ADHD as a cause of behaviors is justified as a direct biological mechanism is believed to underlie the symptoms [16, 17]. Such a framework may warrant simple and certain explanations, whereas the integration of different perspectives requires more nuance and complexity. Furthermore, taking a biomedical approach to ADHD justifies the theory that children with ADHD are distinctly different from other children. A psychosocial approach allows for more normalization of problematic behaviors, specifically for the individual child (13 , 28, 41, 43).

Notably, we found that tensions between biomedical and psychosocial perspectives have resulted in conflict *within* a single thematic stance on ADHD in psychoeducational materials, as opposed to conflict *between* parties with different visions on ADHD. We speculate that these unaddressed, internal conflicts arise from a covert tension within the biopsychosocial model. The biopsychosocial model is one of the most widely accepted approaches to mental health [15]. According to this model the interplay between biological, psychological and social factors underlies behavioral and emotional problems [15, 51]. Within the context of this biopsychosocial model, there seems to be a covert preference for biology [13, 14, 20, 25, 26, 52]. In the materials we analyzed, neurobiological and genetic factors were prioritized: they were discussed ahead of environmental factors, received more attention and were assigned more definitive terminology. In other words: we found a covert *primacy of biology.* This primacy is illustrated in the notion, found in many materials, that ADHD is caused by neurobiological difficulties in the context of environmental risk factors. The opposite was never considered: could ADHD be caused by environmental difficulties in the context of biological risk factors? According to the biopsychosocial model, neurobiological and environmental factors should be considered equally. Yet the ordering and terminology in the materials prioritize biology. This covert *primacy of biology* may well lead to tension in the biopsychosocial model and in turn lead to inconsistent and incoherent information on ADHD.

In sum, we found a number of internal conflicts in how ADHD is framed and contextualized in psychoeducational materials. Notably, these conflicts remained unaddressed in the materials themselves and may potentially lead to confusion, misrepresentation and decontextualization of ADHD. Ultimately, for those diagnosed with ADHD, and their parents, these conflicts may hamper their ability to understand themselves in the context of their attentional difficulties.

### Limitations

A limitation of our study is that we did not involve children with ADHD and their parents. We therefore could not verify that our interpretation of the materials aligns with how parents and children understand and interpret the information. An important next step in this line of research would be to evaluate how psychoeducational materials are interpreted by lay readers.

A second limitation in our study is the use of convenience sampling. We selected the first materials that we came across in our internet search (and that fitted our selection criteria). We chose to select the materials in this way, because we felt that parents of children with ADHD would be most likely to interact with those materials first presented by search engines. However, our sample is not representative of all psychoeducational materials on ADHD. We would have to carry out a much more extensive study to verify such claims. Moreover, we were unable to collect information on funding for psychoeducational materials, as information on websites was often missing or incomplete.

## Supplementary Information

**Additional file 1.** List of Psychoeducational Materials.

**Additional file 2.** Data Analysis Plan.

**Additional file 3.** Examples of internal conflicts in ADHD psychoeducation.

## Data Availability

The materials and texts used and/or analyzed during the current study were found online and can be provided by the corresponding author on reasonable request.
