# Supplementary material for: Lost in explanation: internal conflicts in the discourse of ADHD psychoeducation
Source: BMC Psychiatry. 2022 Nov 8;22:690. doi: 10.1186/s12888-022-04327-x (PMC9644452; doi:10.1186/s12888-022-04327-x)
Supplement: Supplementary file 1 — Additional file 1. List of Psychoeducational Materials. [file 12888_2022_4327_MOESM1_ESM.docx]

**Additional file 3. Examples of internal conflicts in ADHD psychoeducation**

***Pattern 1: Cause vs. Consequence***

| “Attention deficit hyperactivity disorder is a condition, which affects those parts of the brain which control attention, impulses and concentration (a neurobiological condition).” - UK Material 11  “Studies show that ADHD may affect certain areas of the brain that allow us to solve problems, plan ahead, understand others’ actions, and control our impulses.” - US Material 8  “ADHD stands for Attention Deficit Hyperactivity Disorder, which is a chronic psychiatric behavioural disorder that manifests as a persistent pattern of inattention and or hyperactivity-impulsivity that is more frequent and severe than is typically observed in individuals at a comparable level of development.” - UK Material 3  “ADHD can present with different behaviours depending on the age, setting (i.e. school, home, playground) and even motivation (e.g. when doing an activity or something a child likes).” - UK Material 4  “ADHD op volwassen leeftijd kan goed worden behandeld. Het feit dat er een verklaring is voor de jarenlange klachten geeft vaak al opluchting“  [ADHD in adult age can be adequately treated. The fact that there is an explanation for years of complaints usually provides relief] - DU Material 7 | |  |
| --- | --- | --- |
|  | |  |
|  | “ADHD is thought to be caused by an imbalance of two neurotransmitters, dopamine and noradrenaline, which are believed to play an important role in the ability to focus and pay attention to tasks.” - UK Material 11  “ADHD is a common condition that’s caused by differences in the Brain” - US Material 11  “ADHD is geen verklaring voor het gedrag, een kind doet niet druk omdat hij ADHD heeft, maar ADHD geeft een naam aan onrustig, impulsief en onoplettend gedrag“ [ADHD is not an explanation/justification for behavior, a child is not hyperactive because he has ADHD, but ADHD gives a name to restless, impulsive or inattentive behavior] - DU Material 10 | |

|  | |  |
| --- | --- | --- |
|  |  | |

***Pattern 2: Uncertain Complexity vs. Certain Simplicity***

| “Scientists have not yet identified the specific causes of ADHD.” - US Material 2  “ADHD is caused by a complicated combination of multiple factors” - UK Material 1  “Az ADHD okai és kockázati tényezői egyelőre ismeretlenek, de a jelenlegi kutatások szerint a genetika mindenképpen szerepet játszik.”  [The causes and risk factors for ADHD are still unknown, but current research suggests that genetics certainly play a role] - HU Material 4  “Researchers are not sure what causes ADHD.” - US Material 3  “Wat precies de oorzaak van ADHD is, weten we niet“  [We don’t know what exactly the cause of ADHD is] - DU Material 3  “A figyelemhiányos hiperaktivitási zavar okai nincsenek teljesen tisztázva.”  [The causes of Attention Deficit Hyperactivity Disorder are not fully clarified] - HU Material 7  “It's not clear what causes the brain differences of ADHD” - US Material 9 | |  |
| --- | --- | --- |
|  | “ADHD is a disorder in certain areas of the brain and is inherited in the majority of cases. It is not caused by poor parenting or a chaotic home environment” - US Material 7  “Bij mensen met ADHD werkt een bepaald hersengebied namelijk niet goed”.  [In people with ADHD, one particular brain area doesn’t function well] - DU Material 12  “WAT ZIJN DE OORZAKEN VAN ADHD? 75-88% van de onderlinge verschillen is te wijten aan genetische (erfelijke) factoren”  [What are the causes of ADHD? 75-88% of the individual differences can be attributed to genetic (inheritable) factors] - DU Material 1  “Kiemelendő, hogy az ADHD kialakulásához idegrendszeri működésbeli eltérések járulnak hozzá, ezért el kell különíteni azokat az ADHD-hoz hasonlító viselkedési problémákat, melyeket környezeti hatások biztosítanak: mint például nevelési problémák, jelentős iskolai leterheltség, vagy a gyermek életében bekövetkező negatív életesemény."  [It should be emphasized that neurodevelopmental abnormalities contribute to the development of ADHD. It is therefore necessary to distinguish between behavioral problems similar to ADHD that result from environmental effects: such as educational problems, significant school workload, or a negative life events in the child’s life] - HU Material 7  “It is not caused by poor parenting or a chaotic home environment, although the home environment can make the symptoms of ADHD better or worse.” US Material 7  “Brain scan studies and psychological studies have found subtle but distinct differences between the brains of people with and without ADHD, in their structure, the way in which they develop and the ways that they work.”- UK Material 7  “Bij ADHD werken de remmende systemen in de hersenen onvoldoende en hebben de hersenen moeite met het verwerken van informatie.”  [In ADHD the inhibitory systems in the brain work insufficiently and the brain has difficulties with processing information] - DU Material 5 | |

***Pattern 3: Normality vs. Abnormality***

| “Its core symptoms are hyperactivity, impulsivity and inattention. These common childhood behaviours occur on a continuum from normal to abnormal. It can be very difficult to judge what ‘normal’ behaviour is in children; therefore when evaluating children for ADHD, many doctors try to assess the degree of impairment caused by these behaviors.” - UK Material 10  “In de bevolking komen al deze symptomen “dimensioneel” voor. Je kan ze dus meer of minder vertonen, eerder dan wèl of niet. Enkel wanneer een bepaalde ernstgraad wordt overschreden wordt het ADHD genoemd”  [In the population all of these symptoms occur ‘dimensionally’. The extent to which you exhibit them may vary, rather than them being entirely present or absent. Only when a certain level of severity is reached, will it be called ADHD] - DU Material 1  “a figyelemzavar és hiperaktivitás tünetei normális esetben is gyakran előfordulhatnak. Ha valakinek egy izgő-mozgó gyermeke van, akkor laikusként azt gondolhatja, hogy igazából nincs is azzal semmi probléma, hogy valaki gyerekként nagyon aktív vagy éppen sokat szaladgál. Ez valójában egészségesnek is tekinthető egy bizonyos szintig, ám itt mindig a mérték a kérdés.”  [Symptoms of ADHD commonly occur in normal cases too. If someone has a fidgety child, you may think, as a layman, that there is really nothing wrong with being very active as a child or just running around a lot. It can actually be considered healthy to a certain level. But the question here is always to what extent. ] - HU Material 8 | |  |
| --- | --- | --- |
|  | “The recognition of ADHD as a serious medical condition continues to grow by physician groups and government health agencies around the world.” - UK Material 3  “ADHD is a clearly defined clinical condition and not just a label for naughty or badly brought-up children.” - UK Material 7  “Left untreated, ADHD in some children will continue to cause ­serious, lifelong ­problems, such as poor grades in school, run-ins with the law, failed relationships, and the inability to keep a job” - US Material 4  “When ADHD is not treated, it can be hard for kids to succeed. This may lead to low self-esteem, depression, oppositional behavior, school failure, risk-taking behavior, or family conflict.” - US Material 9 | |

***Pattern 4: Specificity vs. Generality***

| “ADHD is a well-defined clinical condition. All the major medical authorities recognise it, including the World Health Organisation and the American Psychiatric Organisation.” - UK Material 7  “ADHD can be categorised by three areas – attention, hyperactivity and impulsivity.” - UK Material 6  ADHD can take different forms in different children but there are three common characteristics which include: Inattention, Hyperactivity and Impulsivity).” - UK Material  “Healthcare professionals use a list of symptoms to officially diagnose ADHD (known as the diagnostic criteria of the American Psychiatry Association DSM-IV or the World Health Organisation ICD10).” - UK Material 11 |  |
| --- | --- |

| **Table 1** | | |
| --- | --- | --- |
| List of all ADHD-related symptoms mentioned in the UK Materials | | |
| Impulsiveness  Hyperactivity/Being overactive  Inattention/ short attention span  Restlessness  Fidgety  Full of energy  Loud and Noisy  Continuous chatter/Talking excessively  Talks when others are talking  Doing things repeatedly without thinking  Finding it hard to wait their turn in games or a queue  Interrupting others in conversation or in play  Hardworking  Persevere at tasks  Eager to try new things  Appear overly forgetful  Distracted  Disorganized | Unable to listen or concentrate  Slow to start tasks  Struggle to finish tasks and often don’t  Creative  Intelligent  Determined  Good at problem-solving  Lack of coordination  Lack of social skills/social clumsiness  Learning difficulties/disabilities  Autism  Conduct disorder/Oppositional defiant disorder  Anxiety  Depression  Dyslexia,  Language problems  Difficulties with handwriting | Neurological problems (tics or epilepsy)  Can't sit still, walks, runs  Can’t do any one thing for very long  Climbs around when others are seated  Daydreaming /seeming to be in another world  Sidetracked by what is going on in surroundings  Mood swings  Being careless  Making too many mistakes at school  Making silly or careless mistakes  Disruptive in play  Always on the go  Often lose their belongings  Lacking attention to details  Being impatient  Poor self-esteem/feeling insecure  Clumsiness  Temper outbursts  Academic underachievement |

***Pattern 5: Necessity of the expert view***

“The knock-on effects of poorly managed or even unidentified ADHD, most notably the potential decline into the criminal justice system, highlight that early intervention is essential.” - UK Material 3

“Left untreated, ADHD in some children will continue to cause serious, lifelong problems, such as poor grades in school, run-ins with the law, failed relationships, and the inability to keep a job.” - US Material 4

“Het voor ADHD zo kenmerkende levenslange patroon van klachten en mislukkingen moet in kaart worden gebracht”

[The lifelong pattern of complaints and failures that is so characteristic of ADHD needs to mapped] - DU Material 7

„Későbbi életkorban egyre gyakoribb az agresszív cselekedet, drog- és alkoholfogyasztás, közlekedési balesetek okozása, kisebb-nagyobb bűncselekmények. Az ilyen esetekben elkövetett, meggondolatlan cselekedetek miatt börtönbe került fiatalok több, mint felénél pszichiátriai betegségeket találtak, melynek 90 százaléka ADHD volt. A fiú-lány megoszlási arány: 9:1 a fiúk „javára”

[Later in their life, aggressive actions, drug- and alcohol use, causing traffic accidents, smaller or larger crimes become more frequent. Children sent to prison for these recklessly committed crimes were found to be diagnosed with a psychiatric disorder in half of the cases, 90% of which were ADHD] - HU Material 3

“Az ADHD egy krónikus zavar, mely hozzátartozik gyermekéhez, és okát az agyban kell keresni.”

[ADHD is a chronic disorder, that is part of your child, and the causes need to be searched in the brain.] - HU Material 2

“Some of the challenges that children with ADHD can face and the wider impact on the family, school staff and other children include: difficulties in school and friendships, underperforming in school and engaging in antisocial activities.” - UK Material 1

“Je ADHD kan je in de weg zitten in je dagelijks leven. Je concentratieproblemen, je impulsiviteit en je drukte zorgen voor problemen. Voor jou, maar ook voor de mensen om je heen. Onze behandelaars kunnen, samen met jou, zorgen dat je je weer kunt ontwikkelen.”

[Your ADHD can get in your way in daily life. Your concentration difficulties, your impulsivity and your hyperactivity cause problems. For you, but also for the people around you. Our clinicians can, together with you, ensure that you can start developing again] - DU Material 12

“Ook is aangetoond dat kinderen met ADHD, gedrag uitlokken bij hun ouders dat het ADHD-gedrag nog gaat versterken (dit noemt men een evocatief gen-omgevingsinteractie-effect)”

[It has also been demonstrated that children with ADHD can provoke behavior in their parents that reinforce even more ADHD-related behaviors (this has been called the evocative gene-environment- interaction effect)] - DU Material 1

“Those who receive specialist support plans tailored to their needs, see the benefits in their learning, friendships, employability and life skills as they understand how best to cope and adapt.” - UK Material 1

“Om erachter te komen of een kind ADHD heeft is een gespecialiseerd onderzoek nodig. Dit onderzoek vindt pas plaats als er een duidelijke aanleiding is”

[To find out if a child has ADHD, specialized testing is needed. This testing only takes place if there is a clear reason] - DU Material 5

“De diagnose ADHD wordt gesteld door een medisch specialist: meestal is dit een (kinder- en jeugd)psychiater.”

[The diagnosis ADHD is given by a medical specialist, usually a (child and adolescent) psychiatrist] - DU Material 8

“A child suffering from ADHD needs treatment across all situations where the difficulties occur. This means support and help at home, school, with friends and community.” - UK Material 4

“A child will need the right treatment and support to ensure they are able to make the most of their education and life in the long-term” - UK Material 5

“However, early identification so that treatment (whether behavioural, psychological or medication) can be started is very important, so that the child is able to achieve his/her full potential”- UK Material 11
