# Supplementary material for: Lost in explanation: internal conflicts in the discourse of ADHD psychoeducation
Source: BMC Psychiatry. 2022 Nov 8;22:690. doi: 10.1186/s12888-022-04327-x (PMC9644452; doi:10.1186/s12888-022-04327-x)
Supplement: Supplementary file 2 — Additional file 2. Data Analysis Plan. [file 12888_2022_4327_MOESM2_ESM.docx]

# **Additional file 1. List of Psychoeducational Materials**

| **Table 1.** | | | | | | | | |
| --- | --- | --- | --- | --- | --- | --- | --- | --- |
| *Materials from the United States* | | | | | | | | |
| **#** | **Type of material** | **Title** | **Author** | **Institution/ Publisher** | **Published in** | **Audience** | **Word Count** | **Link** |
| 1 | website | Attention-deficit/ hyperactivity disorder (ADHD) in children | Mayo Clinic Staff | Mayo Clinic | 2019 | adults | 3298 | <https://www.mayoclinic.org/diseases-conditions/adhd/diagnosis-treatment/drc-20350895> |
| 2 | website | What is ADHD? | Physician Review: Ranna Parekh, M.D. | American Psychiatric Association (APA) | 2017 | adults | 1455 | <https://www.psychiatry.org/patients-families/adhd/what-is-adhd> |
| 3 | website | Attention-Deficit/ Hyperactivity Disorder | National Institute of Mental Health (NIMH) | National Institute of Mental Health (NIMH) | 2019 | adults | 2617 | <https://www.nimh.nih.gov/health/topics/attention-deficit-hyperactivity-disorder-adhd/index.shtml> |
| 4 | website | Understanding ADHD: Information for Parents | American Academy of Pediatrics | American Academy of Pediatrics | Written in 2007.  Last updated in 2019. | parents | 894 | <https://www.healthychildren.org/English/health-issues/conditions/adhd/Pages/Understanding-ADHD.aspx> |
| 5 | website | ADHD – Attention Deficit Hyperactivity Disorder in Northern New Jersey | Psycho-Educational Associates | Psycho-Educational Associates | Not mentioned | parents | 515 | <http://www.psycho-edassociates.com/disorders/attention-deficit-hyperactivity-disorder-adhd-in-new-jersey/> |
| 6 | website | What is ADHD? | Centers for Disease Control and Prevention | Centers for Disease Control and Prevention | Last reviewed in 2021 | parents | 1021 | <https://www.cdc.gov/ncbddd/adhd/facts.html> |
| 7 | website | Parenting a Child with ADHD | Children and Adults with Attention-Deficit/Hyperactivity Disorder (CHADD) | Children and Adults with Attention-Deficit/Hyperactivity Disorder (CHADD) | 2018 | parents | 1530 | [https://chadd.org/wp-content/uploads/2018/03/aboutADHD.pdf](https://chadd.org/for-parents/overview/) |
| 8 | website | What is ADHD? | American Academy of Child and Adolescent Psychiatry (AACAP) | American Academy of Child and Adolescent Psychiatry (AACAP) | Not mentioned | parents | 623 | <https://www.aacap.org/AACAP/Families_and_Youth/Resource_Centers/ADHD_Resource_Center/ADHD_A_Guide_for_Families/What_is_ADHD.aspx> |
| 9 | website | ADHD: What is ADHD | Nemours KidsHealth. Reviewed by: Shirin Hasan, M.D. | Nemours KidsHealth | Last reviewed in 2020 | parents | 963 | <https://kidshealth.org/en/parents/adhd.html> |
| 10 | website | Tell Me All I Need To Know About ADHD | Jennifer Tzeses  Reviewed By: Randy Bressler | Psycom | Last Reviewed in 2021 | adults | 3477 | <https://www.psycom.net/adhd> |
| 11 | website | What is ADHD? | Understood Team | Understood | Not mentioned | youth and adults | 1239 | <https://www.understood.org/en/learning-thinking-differences/child-learning-disabilities/add-adhd/what-is-adhd?_ul=1*1k32del*domain_userid*YW1wLTNzY25OQ1lJa3NTLXNTUjB0ajRtVnc.> |

| **Table 2** | | | | | | | | |
| --- | --- | --- | --- | --- | --- | --- | --- | --- |
| *Materials from the United Kingdom* | | | | | | | | |
| **#** | **Type of material** | **Title** | **Author** | **Institution/ Publisher** | **Published in** | **Audience** | **Word Count** | **Link** |
| 1 | online flyer | Advice and Information for Parents and Carers: ADHD | Young Minds | Young Minds | 2019 | parents | 1293 | <https://youngminds.org.uk/media/3671/adhd-updated-dec-2019.pdf> |
| 3 | website | Q&A What is ADHD | The UK ADHD Partnership (UKAP) | The UK ADHD Partnership (UKAP) | Informationnot provided | adults | 1134 | https://www.ukadhd.com/questions-and-answers.htm?category=112#What%20is%20ADHD? |
| 4 | website | ADHD and hyperkinetic disorder: for parents and carers | Royal College of Psychiatrists | Royal College of Psychiatrists | Information not provided | adults | 2192 | https://www.rcpsych.ac.uk/mental-health/parents-and-young-people/information-for-parents-and-carers/attention-deficit-hyperactivity-disorder-and-hyperkinetic-disorder-information-for-parents-carers-and-anyone-working-with-young-people |
| 5 | website | Attention deficit hyperactivity disorder (ADHD) | The Child and Adolescent Mental Health Service in collaboration with the Child and Family Information Group | NHS - Great Ormond Street Hospital for Children | Last Reviewed in 2016 | adults | 2044 | <https://www.gosh.nhs.uk/conditions-and-treatments/conditions-we-treat/attention-deficit-hyperactivity-disorder-adhd> |
| 6 | website | Attention Deficit Hyperactivity Disorder (ADHD) | Mental Health Foundation | Mental Health Foundation | Information not provided | adults | 543 | <https://www.mentalhealth.org.uk/a-to-z/a/attention-deficit-hyperactivity-disorder-adhd> |
| 7 | online flyer | ADHD: A compact guide for parents | Living with ADHD | Living with ADHD | 2019 | parents | 4012 | https://janssenwithme.co.uk/en-gb/living-with-adhd-parents/adhd-resources |
| 8 | website | ADHD in children and teenagers | Clinical Partners | Clinical Partners | Information not provided | adults | 494 | https://www.clinical-partners.co.uk/child-adolescents/child-teen-adhd/adhd-in-children#what-is-adhd |
| 9 | website | ADHD in children | ADHD Care LTD | ADHD Care LDT | Information not Provided | adults | 540 | https://www.adhdcare.co.uk/?p=adhd.in.children |
| 10 | website | Attention Deficit Hyperactivity Disorder (ADHD) | ADHD Voices | ADHD Voices | Information not Provided | adults | 301 | http://www.adhdvoices.com/adhd/ |
| 11 | website | Information Centre | ADHD Information Services (ADDISS) | ADHD Information Services (ADDISS) | Information not provided | adults | 1774 | http://www.addiss.co.uk/adhd.htm |

| **Table 3.** | | | | | | | | |
| --- | --- | --- | --- | --- | --- | --- | --- | --- |
| *Materials from the Netherlands* | | | | | | | | |
| **#** | **Type of material** | **Title** | **Author** | **Institution/ Publisher** | **Published in** | **Audience** | **Word Count** | **Link** |
| 1 | website | "Psycho-educatie ADHD"  [Psychoeducation ADHD] | "Zorgpad ADHD" [Carepath ADHD] | Zorgpad ADHD  [Carepath ADHD] | 2020 | adults | 3077 | https://www.adhd-traject.be/nl/pagina/adhd |
| 3 | website | "Richtlijn / Info voor Ouders ADHD"  [Guideline / Info for Parents ADHD] | Frits Boer, Barbara van den Hoofdakker, Pier Prins, Wil Hogeman-Weijers, Matthijs Oud Geurt van de Glind & Henny Sinnema | "Richtlijnen jeugdhulp en jeugdbescherming"  [Guidelines for youthaid and youthprotection] | 2016 | parents | 1618 | https://richtlijnenjeugdhulp.nl/adhd/wat-is-adhd/adhd-beloop/ |
| 4 | website | "ADHD: wat werkt? "  [ADHD: what works?] | "Nederlands Jeugdinstituut (NJI) " [Netherlands Youth Institute] | "Nederlands Jeugdinstituut (NJI) " [Netherlands Youth Institute] | Information not provided | adults | 721 | https://www.nji.nl/adhd/wat-werkt |
| 5 | Online Flyer / Website | "Kinderen en druk gedrag"  [Children and hyperactive behavior] | "ADHD wat nu"  [ADHD what now] | "ADHD wat nu"  [ADHD what now] | 2019 | parents | 1378 | https://adhdwatnuweb.nl/ |
| 6 | Online Flyer | "ADHD – Kinderen met aandachts-tekortstoornis met of zonder hyperactiviteit"  [ADHD – Children with Attention Deficit Disorder with or without hyperactivity] | Balans | Balans | Information not provided | parents | 810 | https://balansdigitaal.nl/wp-content/uploads/2021/08/Balans-Folder-ADHD.pdf |
| 7 | Online Flyer | "ADHD bij volwassenen: aandachtstekort-hyperactiviteits stoornis"  [ADHD in adults: Attention Deficit Hyperactivity Disorder] | "Nederlandse Vereniging voor Psychiatrie"  [Dutch Psychiatry Association] | "Nederlandse Vereniging voor Psychiatrie"  [Dutch Psychiatry Association] | 2002 | adults | 3243 | https://praktijk-hagedoorn.nl/assets/uploads/files/In_gesprek_over_ADHD_bij_volwassenen.pdf |
| 8 | website | "ADHD bij kinderen"  [ADHD in Children] | Hersenstichting  [Brain Foundation] | Hersenstichting  [Brain Foundation] | 2020 | adults | 282 | https://www.hersenstichting.nl/hersenaandoeningen/adhd-bij-kinderen/ |
| 10 | Online flyer | "Onrustig, impulsief of onoplettend; ADHD of iets anders? "  [Restless, impulsive or inattentive: ADHD or something else?] | "Monique Verburg, Sanne te Meerman en Leden van de Academische Werkplaats voor ADHD en Druk Gedrag"  [Monique Verburg, Sanne te Meerman and Members of the Academic Workplace for ADHD and hyperactive behavior] | "Academische Werkplaats voor ADHD en Druk Gedrag"  [Academic Workplace for ADHD and hyperactive behavior.] | 2019 | parents | 2435 | https://www.karakter.com/assets/uploads/Downloads/Brochure20voorlichting20versie2011.pdf |
| 11 | website | ADHD | Youz | Youz | Information not provided | adults & children | 2250 | https://www.youz.nl/adhd |

| **Table 4** | | | | | | | | |
| --- | --- | --- | --- | --- | --- | --- | --- | --- |
| *Materials from Hungary* | | | | | | | | |
| **#** | **Type of material** | **Title** | **Author** | **Institution/ Publisher** | **Published in** | **Audience** | **Word Count** | **Link** |
| 1 | website | Hiperaktivitás -Olyan, mint akit felhúztak-  [Hyperactivity- As if they were wound up] | Dr. Garas Péter & Vidomusz Réka | Vadaskert Child and Adolescent Psychiatric Clinic  &  Fitt Békés- Békési egészség monitoring rendszer | 2015 | adults | 650 | <http://www.bekesmegyeiegeszseg.hu/tudastar/cikk/471>  <https://vadaskert.hu/hiperaktivitas/> |
| 2 | booklet | tájékoztató füzet ADHD-s gyermekek szülei részére  [Booklet for parents of children with ADHD] | lilly szülőknek  [Lilly for parents] | Ignáci pedagógiai műhely  [Ignác Pedagogical Workroom] | 2015 | adults | 790 | <http://ignacipedagogia.hu/wp-content/uploads/2017/03/ADHD-T%C3%A1j%C3%A9koztat%C3%B3-f%C3%BCzet-ADHD-s-gyermekek-csal%C3%A1djai-r%C3%A9sz%C3%A9re_hajtogat%C3%B3s.pdf> |
| 3 | website | „Amikor nem járni tanul meg, hanem rohanni először, és amint megtanul beszélni, be nem áll a szája” -avagy mi áll az ADHD hátterében?  [„When they don’t learn to walk, but run first. And when as soon as they learn to speak, they don’t shut their mouth”- or what is behind ADHD? ] | Sófi-Ősz Veronika | Heim Pál Children's Hospital | 2018 | adults | 819 | <https://gyogyhirek.hu/figyelemkontroll-adhd/> |
| 4 | Magazine article | ADHD hipergyerekek  [ADHD hyperchildren] | Bárnosi Eszter | Vadaskert Child and Adolescent Psychiatric Clinic | 2019 | adults | 1259 | <http://vadaskert.hu/wp-content/uploads/2019/03/gyerekle%CC%81lek_adhd.pdf> |
| 5 | website | Figyelemzavaros hiperaktivitás gyermekkorban (ADHD)  [Attention deficit hyperactivity in childhood] | Egészségkalauz  [Health Guide] | Egészségkalauz  [Health Guide] | 2015 | adults | 1112 | <https://www.egeszsegkalauz.hu/betegsegkereso/mentalis-betegsegek-viselkedeszavarok/figyelemzavaros-hiperaktivitas-gyermekkorban-adhd/38mqj42> |
| 6 | website | ADHD  [ADHD] | Virág Henrietta | Családinfó  [Family info] | 2019 | adults | 1228 | <https://csaladinfo.hu/2019/11/adhd/> |
| 7 | website | Hiperaktivitás, ADHD  [Hyperactivity, ADHD] | Budai Egészségközpont  [Buda Health Center] | Budai Egészségközpont Kft  [Buda Health Center] | Information not provided | adults | 788 | <https://bhc.hu/betegsegek/hiperaktivitas/> |
| 8 | website | ADHD, avagy Figyelemhiányos hiperaktivitás zavar  [ADHD, or Attention Deficit Hyperactivity Disorder] | Arbor Egészségfejlesztő központ  [Arbor Health promotion Center] | Arbor Egészségfejlesztő központ  [Arbor Health promotion Center] | Information not provided | adults | 1484 | <http://www.arborek.hu/adhd-avagy-figyelemhianyos-hiperaktivitas-zavar> |
| 9 | website | ADHD  [ADHD] | Csíky Miklós | ADHDoki  [ADHDoc] | Information not provided | adults | 1653 | <http://adhdoki.blogspot.com/p/adhd.html> |
| 10 | website | Az ADHD/ADD-ról részletesen  [About ADHD/ADD in detail] | ADHD Központ  [ADHD Center] | ADHD Központ  [ADHD Center] | Information not provided | adults | 1786 | <https://www.adhdkozpont.hu/az-adhd-rol/> |
