# Supplementary material for: Lost in explanation: internal conflicts in the discourse of ADHD psychoeducation
Source: BMC Psychiatry. 2022 Nov 8;22:690. doi: 10.1186/s12888-022-04327-x (PMC9644452; doi:10.1186/s12888-022-04327-x)
Supplement: Supplementary file 3 — Additional file 3. Examples of internal conflicts in ADHD psychoeducation. [file 12888_2022_4327_MOESM3_ESM.docx]

**Additional file 2.** Data analysis plan

We (RS, DR & MvL) designed an analysis plan and carried out a practice analysis on two Canadian materials. We then rated the British-English materials simultaneously in order to reach further consensus on methodology and findings. Subsequently we rated the American-English (DR), Hungarian (RS) and Dutch (MvL) materials independently. These are the steps taken during the analysis of the materials from each of the countries.

1. **Logging of preconceived notions**
   1. We independently logged our preconceived notions.
2. **Reading of the materials**
   1. We carried out a detailed reading of the materials four times over.
      1. We read each of the 10 materials twice in the following order: 1, 2, 1, 2, 3, 4, 3, 4, 5, 6, 5, 6, 7, 8, 7, 8, 9, 10, 9, 10. The reading of the materials was spread across two days in order to ensure that we were able to stay focused.
      2. During and after the reading of the materials, we kept notes of our thoughts and findings. We also discussed our first impressions.
      3. Without looking at the materials again we wrote down what we remembered from the contents of the materials title by title. We had a short discussion meeting about what we remembered.
      4. We read through the materials again in the same order. We continued to keep notes and had another discussion meeting at the end.
3. **Coding of the materials**
   1. We coded two materials a day. Coding was split up into three steps. We went through these steps for each material consecutively.
      1. *Content coding.* In the first step we strictly coded the content of the material to establish an overview of the information included in the materials.
      2. *Interpretative coding.* The second step involved a more critical interpretation of the data. We coded how the diagnosis ADHD was explicated and framed, how meaning was given to concepts and how implicit ideas and understanding of ADHD were transferred through the text.
      3. *Linguistic coding.* In the third step we coded for language use, wording and phrasing in the text. We did not code all language use in the text, but focused on those phrases that stood out and referred back to the interpretative coding of step 2.
   2. We had daily discussion meetings to talk about the two previously coded materials. We took some time to talk about our findings, how we coded the text and what stood out to us most.
4. **Structuring of the coding**
   1. After the coding of the text, we individually went through our coding and structured our findings. In this individual evaluation we answered the following three questions:
      1. What codes in the content coding did you use most frequently and how can we best summarize and generalize the content of the ten materials? Give an accurate general description of the content of the materials.
      2. What are the most relevant/important/noticeable codes in the interpretative coding? If you could compliment or critique the materials we have read so far, what would you want to point out?
      3. What are the most noticeable linguistic features in the text and how do they link to the interpretative findings? What are the text quotes and examples that best represent the established interpretation in the previous question?
5. **Discussion meeting**
   1. We had an extensive discussion meeting. In this meeting we presented how we individually answered the questions in step four. After we each presented our individual evaluation of the coding, we then found consensus on how to summarize and interpret our findings. We came to a conclusion about the most relevant patterns in the data and how these patterns constructed the information and explanatory frameworks being shared with parents and children.
6. **Preliminary result section**
   1. We composed a preliminary version of our results, in which we wrote down our findings. We linked these to the most relevant text quotes. This preliminary version was then critically discussed with BvH who had deliberately not read the materials at this point in time. This was to warrant that critique was aimed at clarity and not content of the discursive patterns. We made adjustments to the preliminary results accordingly.

After completing these steps for the British English materials, we carried them out independently for the American English, Dutch, Hungarian materials. We had intermittent discussion meetings, but as we were all coding different materials, these meetings were less detailed. After all materials were coded, analyzed and interpreted, we had overarching discussion meetings to evaluate our findings and discuss differences between the languages. These findings were integrated in the preliminary result section.

**Quantitative description**

After defining the major patterns in the data, we reanalyzed the materials to check our findings and count the number of occurrences in the data. This analysis consisted of the following steps:

We defined how to count/code our themes

We piloted these definitions on one material from each language

We analysed the British English materials by coding

We discussed how to count each theme and reached consensus on the occurrences

We individually analyzed the other languages

We discussed any uncertainties and reached consensus on the occurrences within and across materials. We integrated these findings in the result section.
